# Regulation of Glycoprotein VI-Dependent Platelet Activation and Thrombus Formation by Heparan Sulfate Proteoglycan Perlecan

**DOI:** 10.3390/ijms241713352

**Published:** 2023-08-28

**Authors:** Isabella Provenzale, Ilaria De Simone, Jonathan M. Gibbins, Johan W. M. Heemskerk, Paola E. J. van der Meijden, Chris I. Jones

**Affiliations:** 1 Department of Biochemistry, Cardiovascular Research Institute Maastricht (CARIM), Maastricht University, 6229 ER Maastricht, The Netherlands; 2 Institute for Cardiovascular and Metabolic Research (ICMR), School of Biological Sciences, University of Reading, Reading RG6 6EX, UK; 3 Synapse Research Institute Maastricht, Kon. Emmaplein 7, 6217 KD Maastricht, The Netherlands

**Keywords:** collagen-related peptide, endorepellin, glycoprotein VI, perlecan, platelet spreading, proteoglycan

## Abstract

Proteoglycans form a heterogeneous family of proteins with covalently bound sulfated glycosaminoglycans. The extracellular matrix proteoglycan perlecan has been proposed to bind to the platelet- and megakaryocyte-specific receptor G6bB, co-regulating platelet glycoprotein VI (GPVI) signaling. The derived non-sulfate proteoglycan endorepellin was previously shown to enhance platelet adhesion via the collagen receptor, integrin α2β1. Here, we compared the roles of perlecan and other matrix proteoglycans in platelet responses and thrombus formation. We used multi-color flow cytometry to measure the degranulation and integrin αIIbβ3 activation of washed platelets in response to various proteoglycans and collagen-related peptide (CRP), the GPVI agonist. Perlecan, but not endorepellin, enhanced the CRP-induced activation of platelets in a time- and concentration-dependent manner. Similar to collagen, immobilized perlecan, but not other proteoglycans, supported static platelet adhesion and spreading. In-flowed whole-blood perlecan diminished shear-dependent platelet adhesion, while it enforced GPVI-dependent thrombus formation—to a larger extent than endorepellin—to induce more contracted aggregates of activated platelets. We concluded that the sulfated proteoglycan perlecan enhances GPVI-dependent platelet responses extending to thrombus formation, but it does so at the expense of reduced adhesion of platelets under flow.

## 1. Introduction

The extracellular matrix, formed by endothelial and other vascular cells, provides an important regulatory interface between the blood and the vessel wall at sites of endothelial retraction, apoptosis, or damage [1]. The composition of the vascular extracellular matrix can be profoundly changed by pathological conditions such as inflammation and atherosclerosis [2,3]. In recent years, it has become clear that the matrix composition in terms or glycoproteins and lipids also determines the difference between thrombosis-prone and thrombosis-resistant atherosclerotic plaques [3,4,5]; for instance, proteoglycan-rich and lipid- and macrophage-poor atherosclerotic plaques are more sensitive to plaque erosion and thrombus induction. It has been postulated, but not shown, that the abundant presence of negatively charged sulfated proteoglycans in the extracellular matrix play a regulatory role in thrombotic events due to endothelial rupture or erosion when exposed to the flowing blood [3,4].

Both histochemical and proteomic approaches have pointed to a high abundance of distinct proteoglycans in plaques that were collected from human or mouse atherosclerotic arteries [6,7,8]. Based on these studies, we hypothesized that the composition of these proteoglycans plays a role in the thrombogenesis upon plaque rupture or erosion by modulating the interaction of blood platelets with vascular collagens. It has been reported that decorin (a chondroitin and dermatan sulfate proteoglycan) promotes platelet adhesion via the collagen receptor integrin α2β1, an effect that was confined to the protein core devoid of glycan and sulfated side chains [9]. In platelets adhering to decorin, an increased tyrosine phosphorylation of signaling proteins was noted. Biglycan (a small leucin-rich proteoglycan) was found to enhance collagen-dependent platelet spreading and thrombus formation via the signaling collagen receptor, glycoprotein VI (GPVI) [10]. Other authors reported that the proteoglycan perlecan, enriched in heparan sulfate side chains, can reduce platelet adhesion and thrombus formation due to the anti-adhesive effects of the negatively charged side chains [11]. It was also found that the heparan sulfates in perlecan can bind to the platelet-inhibiting G6bB receptor, resulting in a downregulated platelet activation via GPVI [12]. On the other hand, endorepellin, i.e., a non-sulfated derivative of perlecan, was found to support platelet adhesion to collagen via the integrin α2β1 receptor, triggering a signaling enforcement through Src-family kinases [13]. A structurally related glycoprotein is endostatin, which is generated from heparan sulfate-containing collagen XVIII by the protease elastase [14,15,16].

Considering the known cooperative roles of GPVI and integrin α2β1 in collagen-dependent platelet adhesion and thrombus formation [17,18], in this report, we systematically investigated how the best known matrix-derived proteoglycans—perlecan, endorepellin, decorin, and endostatin—regulate the adhesion and activation of platelets mediated by GPVI. Our results pointed to a dual modulating role of perlecan’s core structure on platelet properties, with likely relevance for understanding atherothrombosis.

## 2. Results

### 2.1. Perlecan Enhances GPVI-Induced Platelet Activation

To compare the modulating roles on platelets, we investigated the effects of four proteoglycans present in the pathological extracellular matrix. Criteria for proteoglycan selection were as follows: (i) literature evidence of expression in the subendothelial matrix; (ii) presence or potential accumulation in human atherosclerotic plaques; (iii) expression of relevant side chains (heparan, dermatan or chondroitin sulfates); and (iv) prior evidence on modulation of platelet activation. This resulted in the panel of decorin (with dermatan- and chondroitin sulfate side chains), perlecan (rich in heparan sulfates), endorepellin (perlecan core structure lacking sulfated chains), and the heparan sulfate proteoglycan endostatin. Initial immuno-staining, using selective antibodies, confirmed that both perlecan and endorepellin were present in the extracellular matrix of cultured human vascular endothelial cells (Figure S1).

First, we aimed to establish the mechanism determining whether and how the binding of these proteoglycans would alter platelet activation properties without the interference of plasma. For this purpose, we performed flow cytometry of washed platelets, collected as to retain their high responsiveness upon collagen, thrombin, and ADP receptor stimulation [19,20]. Due to (genetic) inter-subject variance in the platelet GPVI expression levels and activity [21], the proper CRP concentration needed to be determined per donor. Using flow cytometry, we first determined, per donor, the intermediate CRP concentration leading to 40–60% of integrin αIIbβ3 activation and P-selectin expression. As we have shown before, this dose-finding made it possible to identify enhancing effects of priming and secondary agonist [19,20]. Using a range of 0.5–5.0 µg/mL CRP, we thus determined the agonist concentration for intermediate and full responses (Figure S2).

**Figure 1:**
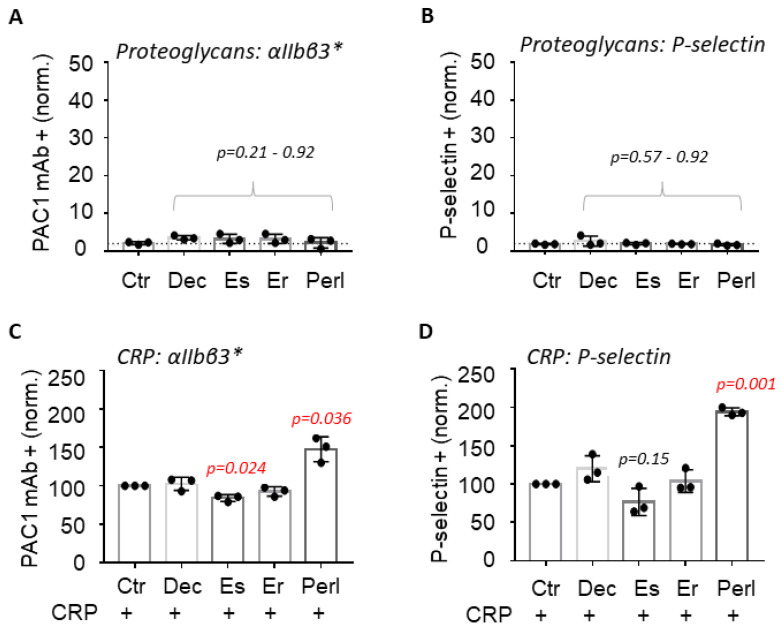
Effect of proteoglycans on CRP-induced platelet activation. Washed human platelets were preincubated with indicated proteoglycan for 10 min and then stimulated by an intermediate concentration of CRP, previously established to induce a 40–60% increase in activation markers. Assayed conditions included vehicle control (Ctr), decorin (Dec, 5 μg/mL), endostatin (Es, 5 μg/mL), endorepellin (Er, 5 μg/mL), and perlecan (Perl, 10 μg/mL). At 10 min after stimulation, activated platelet integrin αIIbβ3* and P-selectin expression were measured via flow cytometry. Raw data of % positive platelets were normalized per experiment versus the control condition with CRP, set at 100. (**A**,**B**) Shown are the normalized values of platelets staining positively with FITC-PAC1 mAb or anti-P-selectin (α-CD62P) mAb in response to proteoglycan alone. (**C**,**D**) Normalized values of platelet staining positively after stimulation with CRP. Means ± SD (*n* = 3); statistical significance for each proteoglycan vs. the control condition is shown in red (1 sample *t* test); non-significance is indicated in black.

At the highest achievable proteoglycan concentration of 5–10 µg/mL, none of the four selected proteoglycans was able to induce platelet integrin αIIbβ3 activation or P-selectin expression by itself (Figure 1A,B). Subsequently, the proteoglycans were combined with the intermediate CRP concentration. Only perlecan enhanced the CRP responses of αIIbβ3 activation and P-selectin expression (Figure 1C,D). The other tested proteoglycans were without effect, with the exception of endostatin, which showed a minor insignificant decrease in CRP-induced integrin αIIbβ3 activation. Additional experiments indicated that perlecan did not affect platelet responses at intermediate concentrations of the PAR1 agonist TRAP6 or ADP (Table S1), suggesting a selective modulation of GPVI receptor signaling.

### 2.2. Perlecan Induces Platelet Spreading

**Figure 2:**
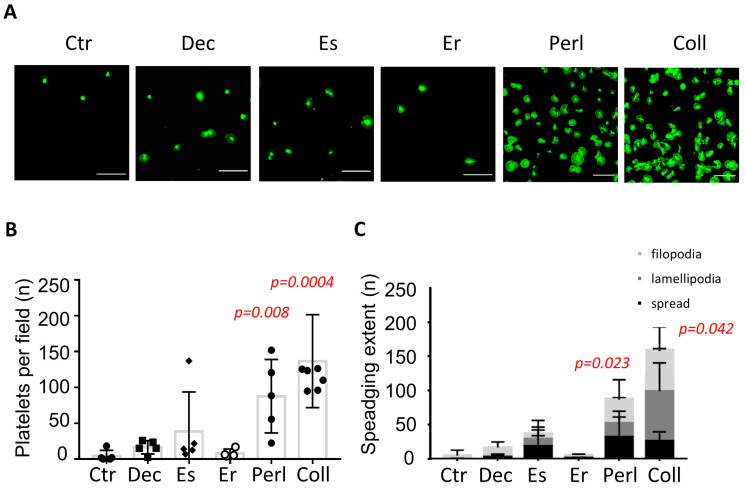
Immobilized perlecan induces platelet adhesion and spreading. Washed human platelets were allowed to adhere and spread on a proteoglycan or collagen-coated glass coverslip for 45 min at 37 °C, fixed, and then stained with DiOC_6_ (green). Control coverslips (Ctr) were treated with BSA buffer. Abbreviations: Dec, decorin; Es, endostatin; Er, endorepellin; Perl, perlecan; Coll, collagen-I. (**A**) Representative images of adhered and spreading platelets. Scale bars, 20 μm. Quantification of numbers of adhering platelets per image field (**B**), and of the extent of platelet spreading: filipodia, lamellipodia, or full spreading (**C**). Means of raw data ± SD (*n* = 5); statistical significance versus control is shown in red via Kruskal–Wallis test (**B**) or two-way ANOVA (**C**).

It was reported that the heparan sulfates on perlecan have an anti-adhesive effect on platelets [12]. To study this further, we coated each of the four proteoglycans on glass coverslips and then determined the adhesion and spreading of washed platelets under static conditions during 45 min. Uncoated, BSA-blocked coverslips and collagen-coated coverslips were used as negative and positive controls, respectively. In the long-term static experiment, only the immobilized perlecan induced substantial platelet adhesion in a manner similar to collagen (Figure 2A). Quantification of the extent of platelet adhesion and platelet spreading pointed to a significant stimulation by the perlecan coating (Figure 2B). Similar to collagen, a proportion of the adhered platelets developed filopodia, lamellipodia, or were fully spread (Figure 2C). The other coated proteoglycans, i.e., decorin, endostatin, and endorepellin, caused no more than limited platelet adhesion. In addition, these proteoglycans essentially failed to induce spreading of the few adhered platelets.

### 2.3. Dual Modulating Effect of Perlecan in GPVI-Dependent Thrombus Formation

**Figure 3:**
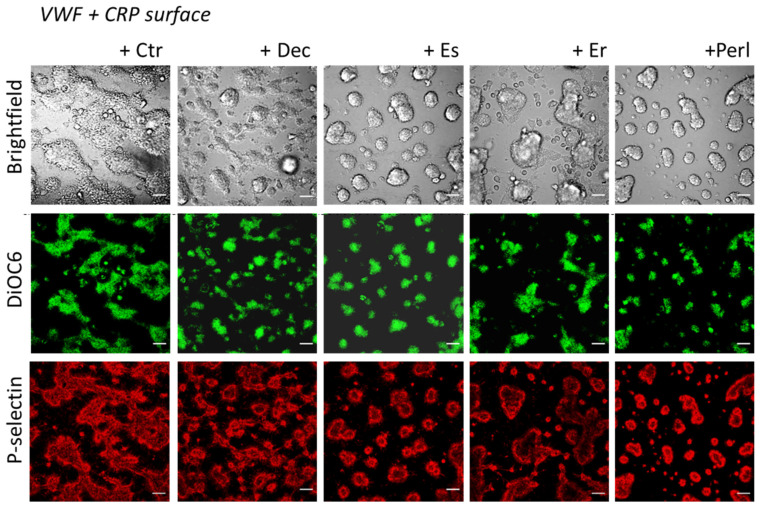
Effects of co-coated proteoglycans on flow-dependent thrombus formation on VWF and CRP. Whole blood was perfused at a shear rate of 1000 s^−1^ through microfluidic channels with microspots of VWF/CRP alone or microspots co-coated with indicated proteoglycans. Abbreviations: Dec, decorin; Es, endostatin; Er, endorepellin; Perl, perlecan. Platelets in blood were stained with DiOC_6_ (green), and thrombi formed on microspots were post-stained for P-selectin expression (AF647 α-CD62P mAb, red). Shown are representative brightfield and confocal fluorescence images of the platelet aggregates after 3.5 min of blood perfusion. Scale bars, 20 μm. Note the smaller sized and more contracted thrombi especially in the presence of perlecan.

As a next step, we used a previously developed microfluidic assay using coating microspots [18] to evaluate the activation-modulating effects of proteoglycans under whole-blood flow conditions. For comparison with the flow cytometric results, we used microspots containing CRP plus von Willebrand factor (VWF), which surface supports platelet adhesion and activation under shear via GPVI and GP-Ib-V-IX (VWF receptor) [18]. For comparison, each of the proteoglycans was therefore co-coated with CRP plus VWF. Coating doses were optimized as before [18]. Blood samples with DiOC_6_-labeled platelets were perfused over the microspots at a standard high wall shear rate of 1000 s^−1^ for 3.5 min. As expected, confocal fluorescence and brightfield microscopy showed that the control surface (CRP/VWF, no proteoglycan) induced the formation of large thrombi of aggregated platelets with high expression of P-selectin. As apparent from Figure 3, on microspots containing some of the co-coated proteoglycans the thrombi looked smaller, while still consisting of P-selectin-expressing platelets.

**Figure 4:**
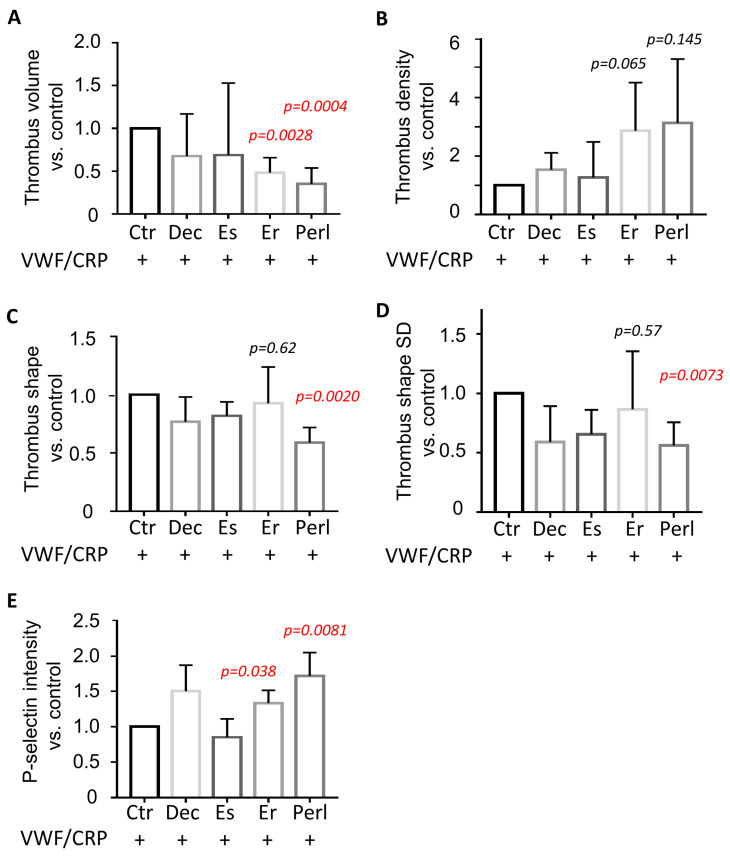
Perlecan alters glycoprotein VI-dependent thrombus structure under flow. Whole blood was perfused through microfluidic channels coated with microspots of VWF/CRP alone or together with indicated proteoglycan at a shear rate of 1000 s^−1^, as for Figure 3. Abbreviations: Dec, decorin; Es, endostatin; Er, endorepellin; Perl, perlecan. Platelets in blood samples were stained with DiOC_6_, and thrombi formed on microspots were post-stained for P-selectin expression. Stacks of raw confocal fluorescence images were analyzed for thrombus structure characteristics (see methods), and data were expressed relative to the day-control condition of VWF/CRP alone (Ctr). (**A**) integrated 3D-DiOC_6_ thrombus volume; (**B**) thrombus density as DiOC_6_ fluorescence intensity per volume; (**C**) mean distance per identified thrombus from centroid to surface; (**D**) standard deviation of distance from centroid to surface, indicative of non-roundness; (**E**) intensity of P-selectin staining, indicative of compactness of activated platelets. Data were normalized to the control condition not containing proteoglycan. Means ± SD (*n* = 3–5 donors), statistical significance versus control in red (1 sample *t* test).

The recording of confocal z-stacks of DiOC_6_-labeled platelets allowed for the quantification of the integrated thrombus volumes on microspots. This analysis pointed to a significant reduction of the thrombi formed on co-coated perlecan or endorepellin (Figure 4A). Calculation of the thrombus density (i.e., the DiOC_6_ fluorescence intensity per volume) showed a significant increase with perlecan and a tendency to increase with endorepellin (Figure 4B). The shapes or sizes of the thrombi were analyzed by measuring the mean distance from a thrombus centroid as well as the standard deviation of this parameter. The obtained values reflect, respectively, the individual thrombus size and the thrombus shape regularity (i.e., the thrombi with lower deviations of the distance from the centroid to the surface are closer to spheroids). Only co-coating with perlecan gave a reduced thrombus shape and a more spheroid structure (Figure 4C,D). In addition, we found that the more contracted thrombi formed on co-coated perlecan or endorepellin had a higher fluorescence intensity for P-selectin expression (Figure 4E), which pointed to a greater accumulation of activated platelets.

**Figure 5:**
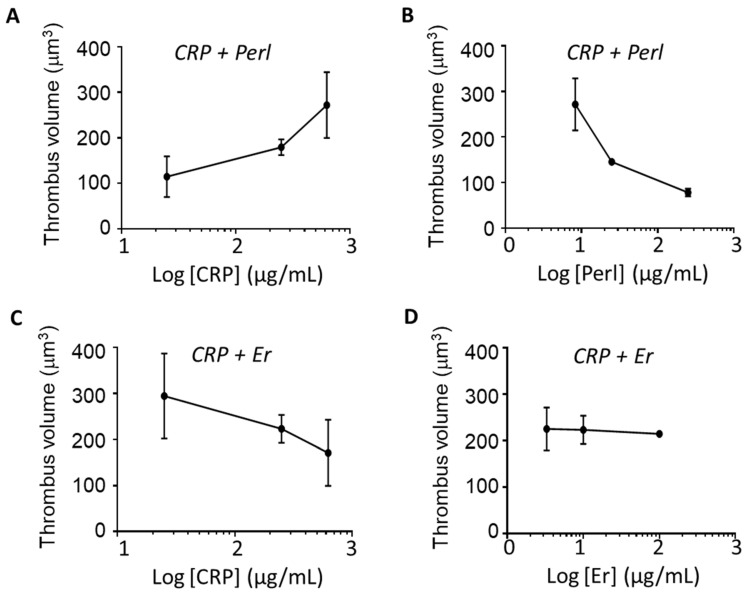
Relative coating concentrations of CRP and perlecan determine whole-blood thrombus formation. Blood with DiOC_6_-labeled platelets was perfused through microfluidic channels coated with 1.5 μL microspots containing a fixed amount of VWF, and different amounts of CRP (25, 250, 625 μg/mL) and perlecan (Perl, 8.3, 25, 250 μg/mL) or endorepellin (Er, 3.3, 10, 100 μg/mL). Blood flow was at a shear rate of 1000 s^−1^ for 3.5 min, with image analysis as for Figure 4. (**A**) Quantification of integrated thrombus volume/field on microspots of perlecan (25 μg/mL) and variable amounts of CRP (25, 250, 625 μg/mL). (**B**) Idem for a fixed concentration of CRP (250 μg/mL) and variable amounts of perlecan. (**C**) Quantification of integrated thrombus volume on microspots of endorepellin (10 μg/mL) and variable amounts of CRP. (**D**) Idem for fixed amount of CRP (250 μg/mL) and increasing amounts of endorepellin. Means ± SD (*n* = 3).

Additional blood flow experiments indicated that the thrombus volume on microspots of VWF, CRP, and perlecan increased with the coated CRP concentration and de-creased with the coated perlecan concentration (Figure 5A,B). However, such coating-concentration-dependent changes were not seen on microspots of VWF, CRP, and endorepellin (Figure 5C,D). This suggested a stronger GPVI-dependent thrombus-modulating role of perlecan than of endorepellin. Further experiments were performed using surfaces of VWF alone with co-coated proteoglycans in order to check for GPVI-independent effects. In this case, the perfusion of blood over VWF plus perlecan or endorepellin resulted in a reduction of the formed micro-thrombi, when compared to the VWF coating alone (Figure S3A). Quantification of the integrated volumes of the formed micro-thrombi (from confocal z-stacks of DiOC_6_-labeled platelets) indicated a restricted thrombus formation on the surfaces containing either perlecan or endorepellin (Figure S3B). Staining the micro-thrombi for P-selectin, however, did not show an effect of either proteoglycan on platelet activation (Figure S3C). Regarding the other proteoglycans, neither decorin nor endostatin showed a significant reduction in micro-thrombus size (Figure S3B).

Collectively, these results point to different effects of the immobilized proteoglycans. Only perlecan induced long-term platelet spreading under stasis, while under flow, both perlecan and endorepellin interfered with the acute VWF-dependent platelet adhesion and strengthened the GPVI-dependent thrombus buildup of activated platelets.

## 3. Discussion

In this study, we compared the ability of four proteoglycans present in the vascular extracellular matrix to modulate platelet activation and thrombus formation. The selected proteoglycans, differing in sulfated side chains, were as follows: decorin (dermatan and chondroitin sulfate chains), perlecan (heparan sulfated), endorepellin (C-terminal part of perlecan), and endostatin (C-terminal part of heparan-sulfated collagen XVIII). When tested in solution, we observed that none of the proteoglycans per se induced platelet activation, whereas only perlecan was able to enhance the GPVI-induced platelet activation in response to CRP. When immobilized on a surface, only perlecan by itself promoted long-term (autocrine-dependent) platelet adhesion and spreading, thus supporting a positive platelet-regulatory function; on the other hand, in the whole-blood setting of shear- and VWF-dependent platelet adhesion, perlecan, to a larger extent than endorepellin, diminished platelet adhesion; however, regarding the still-adhered platelets, it enhanced GPVI-dependent thrombus formation to result in more contracted thrombi. Together, these findings point to a dual—partly stimulating and partly inhibiting—effect of specifically perlecan, suggesting interference with two different receptors and/or signaling pathways.

Earlier studies on the interaction of platelets with proteoglycans were confined to static or slowly stirred conditions. Herein, in line with the present results, no platelet-adhesive role of decorin was found [9,10,11,12,13]. Regarding endostatin, it is known to be released from platelets and act as an angiogenesis inhibitor [16]. In some studies, perlecan was assigned as a platelet anti-adhesive matrix component, which was lost after removal of the heparan sulfate chains [11]. In other studies, perlecan and the derived endorepellin were found to enhance integrin α2β1-dependent platelet adhesion to collagen [13] and to interact with the platelet-inhibiting tyrosine phosphatase-linked receptor G6bB [12]. Although, in our static experiments, perlecan supported platelet adhesion and spreading more than endorepellin, the results suggest a dual mechanism in which the proteoglycans stimulate integrin α2β1 and/or αIIbβ3 interactions and simultaneously suppress GPVI activity via G6bB signaling. Jointly, such bivalent effects may lead to a re-setting of the integrin-dependent effects on GPVI-induced thrombus formation under flow.

Platelet spreading on surfaces such as fibrinogen or collagens is known to be driven by integrin αIIbβ3 outside-in signaling as well as by GPVI signaling [22,23]. The ability of immobilized perlecan to induce platelet spreading and full lamellipodia formation is hence supportive of a role for signaling by integrin αIIbβ3 and/or GPVI. In other experiments, under conditions of blood flow over collagen, fibrin or fibrinogen, a non-redundant cooperativity was demonstrated in thrombus buildup between GPVI and platelet integrins [24,25]. Our current data suggest that in the presence of proteoglycans, this cooperativity can be altered, although this needs further validation. For the flow cytometric assessment of CRP-induced platelet activation, the stimulating effect of perlecan may also be explained through modulation of integrin-dependent GPVI signaling. A limitation of this analysis is the relatively small number of independent samples (three for the comparison of the CRP-induced activation responses; five for the comparison of platelet adhesion and platelet spreading).

Vögtle et al. reported that the static adhesion of platelets to perlecan increased after digesting its heparan sulfate side chains [12]. In contrast, in our experiments with immobilized proteoglycans, perlecan was more active than endorepellin in inducing platelet spreading, even to an extent comparable to collagen. The previous report proposed a model in which the heparan sulfate side chains of perlecan downregulate GPVI-induced platelet activation through the receptor G6bB [12] such that the tyrosine phosphatase Shp2 (*PTPN11*) generates platelet-inhibiting signaling responses [26]. However, the functional consequences of this interaction were not fully elucidated at the time.

Another novel finding is the dual type of effects of immobilized perlecan—echoed by endorepellin—on the shear- and GPVI-dependent thrombus formation on VWF/CRP surfaces, i.e., a reduction in platelet adhesion but an increase in thrombus contraction. Endorepellin showed a lesser effect on thrombus contraction than perlecan, despite a higher calculated molar coating density (37 pmol/mm^2^, in comparison to 18.5 pmol/mm^2^ for perlecan), thus supporting an additive role for the heparan sulfate groups in perlecan. One explanation is that under the current flow conditions, with a strong GPVI ligand such as CRP, the antagonistic role of G6bB is limited to those platelets not in direct contact with this ligand. Other explanations are that the integrin-enhancing effect of platelets in contact with CRP overcome the G6bB effect, or that the degree of sulfation on perlecan is too low to fully activate G6bB [12].

In addition to controlling GPVI-dependent platelet processes, engagement of the ITIM-bearing G6bB receptor can also co-regulate integrin outside-in signaling [26,27]. Thus, other ITIM-linked receptors on platelets such as PECAM1 and CEACAM1/2 are able to regulate outside-in signaling [28,29]. These types of effects will depend on the nature of the receptor engagement duration, avidity, affinity, and clustering [30]. Overall, our findings are suggestive of a dual feature of perlecan’s core structure—which is also present in endorepellin—to act on one hand as a ligand for platelet integrins and, on the other hand, as a modulating agent of signaling platelet collagen receptors.

## 4. Materials and Methods

### 4.1. Materials

Human von Willebrand factor (VWF) native protein (RP-43132) was obtained from Thermo-Fisher Scientific (Pasley, UK). Cross-linked collagen-related peptide CRP was obtained from CambCol Laboratories (Cambridge, UK). Decorin isolated from bovine cartilage (D8428), human recombinant endostatin (GF171), and perlecan HSPG (H4777) were purchased from Sigma-Aldrich (Rotterdam, The Netherlands). Human recombinant endorepellin (2364ER) was obtained from R&D Systems (Abingdon, UK). Collagen Horm (collagen-H) derived from equine tendon was obtained from Nycomed (London, UK). BSA and hirudin (94581-1EA) were obtained from Sigma-Aldrich (Poole, UK). DiOC_6_ (6975) was obtained from AnaSpec (San Jose, CA, USA). Fluorescein isothiocyanate (FITC)-labeled PAC1 monoclonal antibody (mAb) against activated integrin αIIbβ3 came from BD Bioscience (Franklin Lakes, NJ, USA). AF647-labeled anti-human CD62P mAb was obtained from Biolegend (San Diego, CA, USA). The anti-human endorepellin/perlecan Ab (AF2364-SP) was obtained from R&D systems (Abingdon, UK). The antibody against human heparan sulfate proteoglycan 2 and perlecan (Ab2501) was obtained from Abcam (Cambridge, UK). Secondary antibodies AF488-labeled anti-rat IgG and AF647-Germanylabeled anti-goat IgG were from Life Technologies (Paisley, UK).

### 4.2. Blood Donors and Platelet Isolation

Blood samples were obtained from healthy donors who had given informed consent with procedures approved by the University of Reading Research Ethics Committee. Samples were collected into vacutainers containing 3.8% (*w*/*v*) sodium citrate. Subjects had not used anti-platelet medication for at least 2 weeks. Platelet-rich plasma (PRP) and washed platelets were obtained from citrated blood samples by centrifugation in the presence of 10 vol% of ACD (acid citrate dextrose, 85 mM sodium citrate, 78 mM citric acid, 11 mM glucose) and washed with Hepes buffer pH 6.6 (136 mM NaCl, 10 mM Hepes, 2.7 mM KCl, 2 mM MgCl_2_, 1 mg/mL glucose, 1 mg/mL bovine serum albumin, BSA), similar to before. Platelet pellets were resuspended in Hepes buffer, pH 7.45 (136 mM NaCl, 10 mM Hepes, 2.7 mM KCl, 2 mM MgCl_2_, 1 mg/mL glucose, 1 mg/mL bovine serum albumin). During the centrifugation and washing procedure, 0.2 units/mL apyrase was added to prevent platelet activation by traces of released autocrine ATP and ADP [31].

### 4.3. Flow Cytometry

Flow cytometric analysis of platelet activation was performed, essentially as before [19]. In brief, washed platelets (100 × 10^9^/L) in Hepes buffer pH 7.45 (10 mM Hepes, 136 mM NaCl, 2.7 mM KCl, 2 mM MgCl_2_) were pre-incubated with proteoglycans at indicated concentrations immediately before agonist addition and then stimulated with CRP in the presence of 2 mM CaCl_2_. Directly after agonist addition, platelets were stained for activated integrin αIIbβ3 and P-selectin exposure, using an FITC-conjugated PAC1 mAb (1.25 µg/mL) and an AF647-conjugated anti-P-selectin mAb (2.5 µg/mL), respectively. Platelet samples were measured for the two activation markers after 10 min of agonist addition according to protocols described before [19]. Flow cytometry was performed using a BD Accuri C6 flow cytometer (BD Bioscience, Franklin Lakes, NJ, USA). A minimum of 10,000 events was counted per assay. Platelet treatment with iloprost (10 nM) did not influence the basal fluorescence levels for integrin αIIbβ3 activation or P-selectin expression.

For each platelet preparation (blood donor), at first, an agonist dosing curve was made per fluorescent probe. To assess effects of proteoglycans, an intermediate agonist concentration (range 0.5–5.0 μg/mL CRP) was chosen to obtain 40–60% of platelets positively staining for FITC-conjugated PAC1 mAb and P-selectin (Figure S2), similarly to as described before [32]. Platelet activation experiments were performed at least in duplicate. Effects of proteoglycans were expressed as percentage of positive platelets per activation marker versus the vehicle control condition.

### 4.4. Platelet Spreading

Eight-well glass bottom µ-slides (Ibidi, Munich, Germany) were coated with decorin (100 μg/mL), endostatin (10 μg/mL), endorepellin (10 μg/mL), perlecan (25 μg/mL), or type I collagen-H (100 μg/mL) (1 h, room temperature) and then blocked with 5 mg/mL of heat-denatured bovine serum albumin (1 h, room temperature). Control coverslips remained uncoated and were then blocked. After washing the coated wells three times with phosphate-buffered saline (PBS: 10 mM Na_2_HPO_4_, 1.8 mM KH_2_PO_4_, 2.7 mM KCl and 137 mM NaCl, pH 7.4), washed platelets (150 μL of 1 × 10^8^/mL) in Hepes buffer pH 7.45 were allowed to adhere for 45 min at 37 °C; then, they washed three times with PBS and fixed with 10% formalin solution (Sigma). Subsequently, the fixed cells were washed three times with PBS and stained with the dye DiOC_6_ (2 μM). For wells from each donor, 3 representative microscopic images per coating were taken. Fluorescence images of adherent platelets were captured with the 20× objective lens of a confocal Ti2 microscope (Nikon, Surbiton, UK).

Image analysis was performed by counting the total number of adhered platelets in ImageJ. The extent of platelet spreading was by scoring (visual inspection of morphology) the platelets forming filopodia or lamellipodia, or the platelets with a fully spread shape.

### 4.5. Microfluidic Thrombus Formation

Whole-blood perfusion assays were performed as described before [18]. Comparative runs were performed with glass coverslips, coated with two microspots of (from outlet to inlet) a combination of CRP (0.5 µL, 250 µg/mL) plus VWF (0.5 µL, 12.5 µg/mL), or with VWF alone (0.5 µL, 12.5 µg/mL). Where indicated, the microspots were co-coated with the indicated proteoglycans (maximally effective concentration tested in prior platelet spreading assays). In detail, under standard conditions, the proteoglycan-containing microspots were coated with decorin (100 μg/mL), endostatin (10 μg/mL), endorepellin (10 μg/mL), or perlecan (25 μg/mL). These concentrations provided a calculated molar coating density of 228, 148, 37, and 18.5 pM/mm^2^, respectively. The coated coverslips were incubated for 1 h in a humid chamber at room temperature and then blocked with Hepes buffer (pH 7.45) containing 1% BSA for 30 min. After mounting into a Maastricht microfluidic chamber (width 5 mm, depth 50 μm, length 30 mm), blood perfusion was started. Per flow run, 1 mL of citrated, 10 unit/mL hirudin-anticoagulated whole blood was perfused for 3.5 min at a wall-shear rate of 1000 s^−1^.

Platelets in the blood samples were pre-stained with DiOC_6_ (0.5 μg/mL), and after flow, the formed thrombi were post-stained with AF647-labeled anti-CD62P mAb (1.25 μg/mL), as detailed elsewhere [18]. Before imaging, residual labels were removed by rinsing with Hepes buffer pH 7.45 containing 2 mM CaCl_2_ and 1 unit/mL heparin. Using confocal microscopy, two representative z-stacks were obtained per microspot with a 60× oil objective lens and a Nikon Ti2 fluorescence microscope (Nikon). All flow runs were performed in duplicate. For all conditions, blood was used from at least 3 different donors.

### 4.6. Assessment of Thrombus Parameters

Cumulative thrombus volumes and single-color fluorescence intensities were measured based on manual cut-off for background using the NIS element AR software (version 4.5) of Nikon’s Imaging Software-Elements, package Advanced Research. Thrombus contraction was calculated via the DiOC_6_-staining density and expressed as fluorescence intensity/volume ratios. Other parameters of thrombus morphology (mean distance from centroid to surface with standard deviations) were obtained in Fiji/ImageJ, using the ‘3D objects count’ function [33].

### 4.7. Culturing of Endothelial Cells

Human umbilical vein endothelial cells (Promocell, Heidelberg, Germany) of passage 4 were cultured on eight-well glass bottom µ-slides (Ibidi, Munich, Germany) coated with rat tail collagen (35.5 μg/mL, EMD Millipore, Darmstadt, Germany) in regular endothelial cell growth medium (Promocell C-22010) and supplemented with growth medium supplement mix (Promocell C-39215), 1% penicillin/streptomycin (Gibco, Waltham, MA, USA), and 1% L-glutamine (Gibco). The cells were cultured for seven days, and the medium replaced every other day with freshly prepared sterile ascorbic acid (50 μg/mL, from Sigma, UK)-supplemented medium. After tissue culture, the extracellular matrices were extracted with a solution containing 5% Triton-X-100 and 20 mM NH_4_OH, essentially as described before [34,35,36]. Subsequently, after carefully rinsing three times with PBS, the matrices were fixed with 10% formalin (Sigma) and stained with primary rat, anti-human perlecan antibody (1:100, overnight at 4 °C), followed by AF647-labeled anti-rat IgG (1:200, 1 h at room temperature) and goat, anti-human endorepellin antibody (1:40 μg/mL, overnight at 4 °C), and subsequently followed by incubation with the secondary AF488-labeled anti-goat IgG (1:200, 1 h at room temperature). Control wells were incubated with secondary antibodies only. Cell nuclei were stained with DAPI (10 min at room temperature). All incubations were followed by three PBS wash steps. Fluorescence images were captured with the 60× objective lens of a confocal A1R microscope (Nikon).

### 4.8. Statistical Analyses

Our general statistical hypothesis in all cases was that the proteoglycan of interest had no effect in comparison to the control condition. Raw or normalized (where indicated) effect data were statistically analyzed using GraphPad Prism V.8 software. Single comparisons to control conditions were tested with 1 one-sample *t* test. For spreading assays, where 1 non-normalized parameter and 3 morphological groups were compared, testing for consistency was conducted via one-way or two-way ANOVA, respectively. Statistical significance was set at *p* < 0.05.

## 5. Conclusions

Conclusively, our data indicate that, in particular, the sulfated and matrix-expressed proteoglycan perlecan acts by enhancing the process of GPVI-induced platelet activation. The modulating responses of perlecan extend from isolated platelets to whole-blood thrombus formation, and this in spite of the fact that perlecan—through a different mechanism—interferes with platelet adhesion under flow.

## Data Availability

All data are included in the manuscript as figures, tables, or supplementary figures.

## Supplementary Materials

The following supporting information can be downloaded at: https://www.mdpi.com/article/10.3390/ijms241713352/s1.
