# Supplementary material for: Regulation of Glycoprotein VI-Dependent Platelet Activation and Thrombus Formation by Heparan Sulfate Proteoglycan Perlecan
Source: Int J Mol Sci. 2023 Aug 28;24(17):13352. doi: 10.3390/ijms241713352 (PMC10487520; doi:10.3390/ijms241713352)
Supplement: Supplementary file 1 [file ijms-24-13352-s001.zip › ijms-2533187-supplementary.pdf]

	Plus TRAP6		Without TRAP6	
	**Control**	**+ PTG**	**Control**	**+ PTG**
FITC-PAC1 mAb (TRAP6 intermediate = 100)				
Decorin (5 µg/mL)	100	106.1 ± 6.6	1.4 ± 0.4	2.9 ± 2.2
Endostatin (5 µg/mL)	100	102.3 ± 12.7	1.7 ± 0.3	1.8 ± 0.3
Endorepellin (5 µg/mL)	100	114.9 ± 6.2*	1.8 ± 0.4	1.7 ± 0.7
Perlecan (10 µg/mL)	100	104.9 ± 7.2	1.6 ± 0.2	1.2 ± 0.8
	**Plus ADP**		**Without ADP**	
	**Control**	**+ PTG**	**Control**	**+ PTG**
FITC-PAC1 mAb (ADP intermediate = 100)				
Decorin (5 µg/mL)	100	100.1 ± 8.3	1.8 ± 0.7	2.1 ± 1.3
Endostatin (5 µg/mL)	100	102.1 ± 11.7	2.0 ± 0.5	2.3 ± 1.6
Endorepellin (5 µg/mL)	100	108.1 ± 8.9	2.3 ± 0.6	2.4 ± 0.9
Perlecan (10 µg/mL)	100	105.4 ± 5.1	2.0 ± 0.1	2.6 ± 1.8
	**Plus TRAP6**		**Without TRAP6**	
	**Control**	**+ PTG**	**Control**	**+ PTG**
AF647-αCD62P mAb (TRAP6 intermediate = 100)				
Decorin (5 µg/mL)	100	99.0 ± 13.7	6.6 ± 4.7	8.6 ± 4.4
Endostatin (5 µg/mL)	100	100.2 ± 15.1	3.3 ± 0.5	4.6 ± 2.7
Endorepellin (5 µg/mL)	100	107.2 ± 10.7	2.8 ± 1.0	5.0 ± 4.9
Perlecan (10 µg/mL)	100	102.7 ± 4.9	4.0 ± 4.0	4.0 ± 2.8

Table S1. Effect of proteoglycans on TRAP6- and ADP-induced platelet activation. Washed human platelets were preincubated with indicated proteoglycan (PTG) for 10 min, and then stimulated by an intermediate concentration of TRAP6 or ADP, inducing a 40-60% increase in activation markers. At 10 min after stimulation, activated integrin αIIbβ3* (FITC-PAC1 mAb) and P-selectin expression (AF647-αCD62P mAb) were measured by flow cytometry. Raw data of % positive platelets were normalized per experiment versus the control condition with agonist, set at 100. Shown are normalized values of activated platelets in the absence or presence of agonist and/or PTG. Note that ADP stimulation caused only minimal P-selectin expression. Means ± SD (n = 3), *p= 0.017 versus control (1 sample t-test).

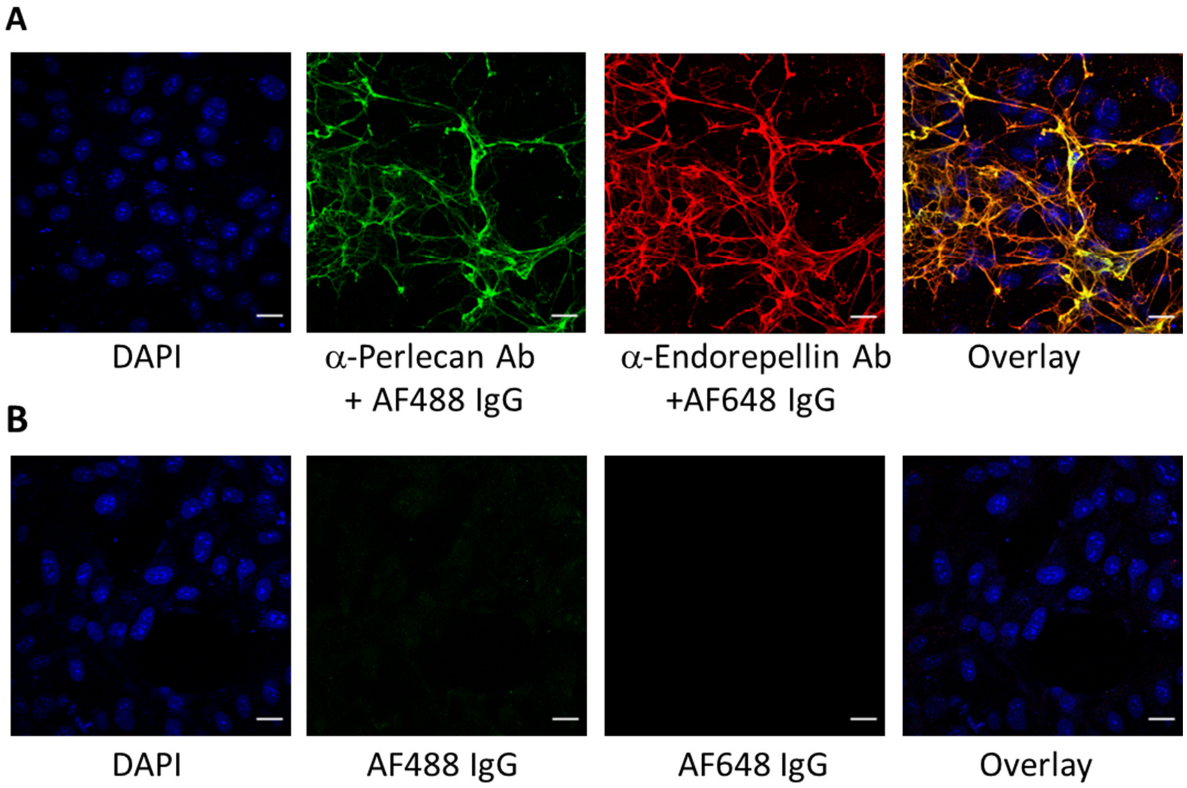

Figure S1. Identification of perlecan and endorepellin in the extracellular matrix from human umbilical vein endothelial cells (HUVEC). The HUVEC were cultured in 12-well plates for 7 days, after which the extracellular matrix was extracted, fixed, and immune-stained for perlecan or endorepellin. Shown are representative confocal microscopic images, indicating staining of endothelial nuclei (DAPI, blue); perlecan (Perl, anti-perlecan mAb + AF647-labeled IgG, green); and endorepellin (Er, anti-endorepellin Ab + AF648-labeled IgG, red). Scale bars, 20 μm.

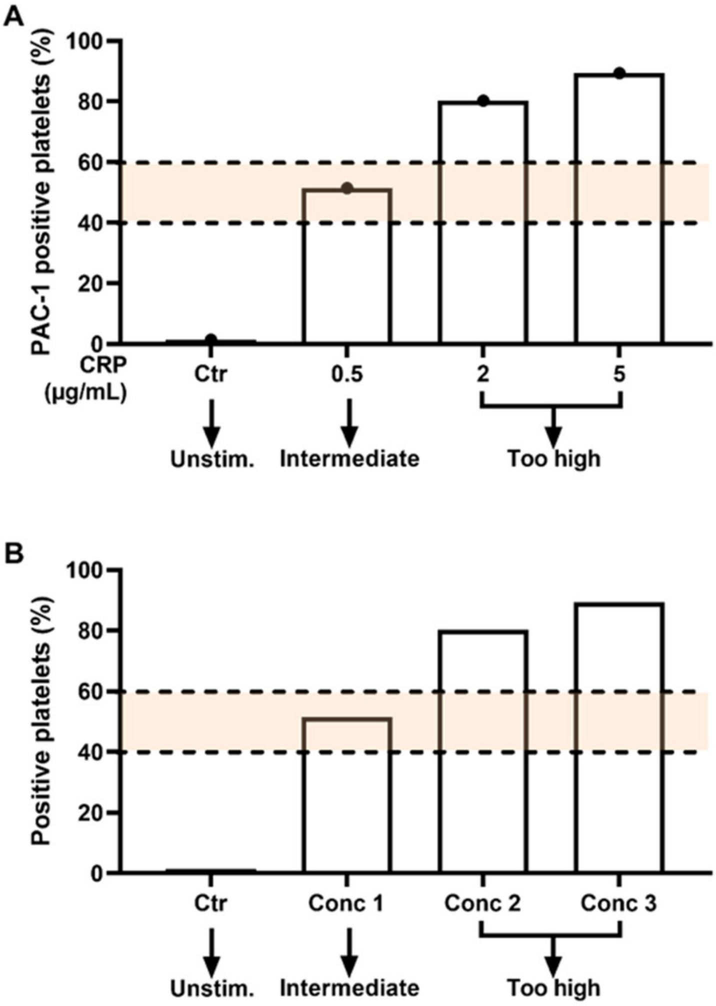

Figure S2. Determination of intermediate agonist concentration for platelet activation. Washed platelets from different donors were stimulated with a range of CRP concentrations (0.5-5 µg/mL) to induce minimal to full activation, as determined by flow cytometry. (**A**) Percentages of CRP-induced FITC-PAC1 mAb positive platelets from a given donor, indicating how the intermediate responsiveness was determined. (**B**) Illustration of concept of agonist titration per donor. First, an unstimulated platelet sample was measured, whereafter the marker for activation was set at 2% positive. Then, effects on FITC-PAC1 mAb mean fluorescence intensity in response to ≥3 agonist concentrations were measured; a concentration inducing a response of 40-60% positive was selected as intermediate for subsequent experimentation.

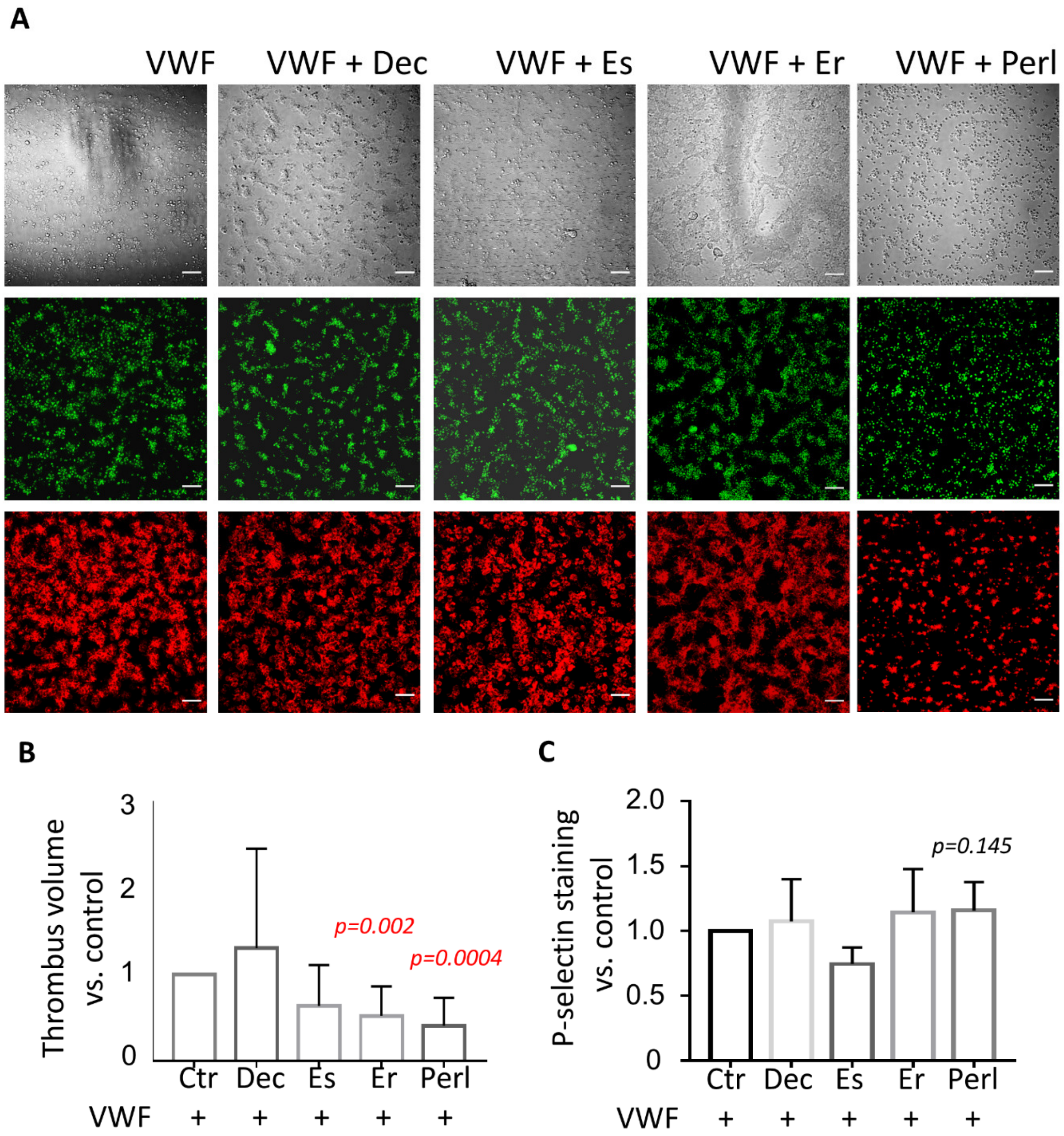

Figure S3. Effect of co-immobilized proteoglycans on VWF-dependent thrombus formation under flow. Whole blood was perfused through microfluidic channels containing microspots of VWF plus indicated proteoglycan at a shear rate of 1000 s^{-1}. Control microspots (Ctr) contained only VWF. Further abbreviations: Dec, decorin; Es, endostatin; Er, endorepellin; Perl, perlecan. (**A**) Representative z-stacks of platelet micro-thrombi formed after 3.5 min of blood perfusion. Platelets were pre-stained with $DiOC_6$ (green), and thrombi were stained post-perfusion for P-selectin expression (AF647 α-CD62P mAb, red). Scale bars, 20 µm. Quantification of micro-thrombus volume (**B**)and P-selectin expression (**C**), relative to the control condition. Means ± SD (n = 3-5 donors), statistical signficiant in red (1 sample t test).
